# Red Clay as a Remedy for Sprains

**Published:** 1884-12-20

**Authors:** G. G. Roy

**Affiliations:** Professor of Materia Medica in the Southern Medical College, Atlanta, Ga.


### RED CLAY AS A REMEDY FOR SPRAINS.

By G. G. ROY, M. D., Professor of Materia Medica in the SOUTHERN MEDICAL COLLEGE, Atlanta, Ga.

The negroes of Virginia would be greatly amazed and astonished if they were to hear that Dr. Thomas L. Shearer had published his "*New* Method of Treating Sprains" in the Lancet, as announced by the Medical and Surgical Reporter of November 15.

Since my earliest recollection the application of red clay, made soft with hot vinegar, has been used as a domestic remedy in Virginia for bad sprains; and the same remedy, made soft with hot water, has likewise been used as an application to inflammation of the eyes where there was great tumefaction of the lids, with general conjunctivitis. And, if I mistake not, the use of this remedy in both instances originated with the negroes, whom it is said learned it from the Indians in the early settlement of the State.

But, because it did thus originate, permit me to say it is not the less efficacious. For severe sprains, it is the very best remedy I have ever seen used. It relieves pain; diminishes redness, swelling, and stiffness of the joint more promptly and effectually than is done by any other mode of treatment that I am cognizant of.

I have seen its application astonish learned physicians with its results, and demonstate the truth of Scripture—that God oftentimes uses the weak things of this world to confound the strong.

Since my residence in this city, I call to mind the case of a brick-mason, who fell some twenty or thirty feet from a chimney he was building to the ground below, and miraculously escaped with only a severely sprained ankle and a few bodily bruises. A physician was summoned, who bandaged the ankle and gave a scientific recipe for a liniment, with which the nurse was instructed to keep the bandage saturated during the night. At his visit next morning he found his patient in great agony, with an enormously swollen, throbbing ankle, from which the man's wife had cut the bandage during the night. He directed the limb to be elevated and the application of the liniment continued. But the poor man's wife thought she knew a better remedy—and she did. She sought the bowels of Mother Earth, from which she dug out a few handsful of red clay, and having thoroughly moistened and softened it with hot vinegar, she enveloped the foot, ankle, and lower half of the leg with this poultice, and upon the arrival of the doctor next day all redness, swelling, pain, and stiffness had disappeared, and his patient reported that he felt about well enough to go to work. The doctor was delighted and astonished at the great improvement, and was praising the wonderful virtues of his liniment, when he was told by the wife—to his greater astonishment—that a red clay poultice had done it all !

I have in mind another case—that of a fashionable young lady of this city, who, in jumping from a carriage, received a very severe sprain of the foot and ankle. Having reached her bedside after another physician had gotten there, I suggested to him to try the clay and vinegar poultice ; but the remedy was too simple, and repugnant to the taste of a fashionable young lady, who, I dare say, belonged to that class of fastidious people we sometimes meet with who had rather suffer, and even die, under the use of elegant remedies than to be cured by Nature's rude and simple appliances. At any rate, my suggestion was rejected ; and when I heard from the lady, three or four weeks afterwards, she was still confined to her bed, with her ankle bandaged, and the liniment her constant companion.

In conclusion, I wish to record, most cheerfully, my full and complete endorsement of Dr. Shearer's "New Method of Treating Sprains"—and so will all the negroes, and the doctors too, at least of Eastern Virginia.

